# Effect of adding the novel fiber, PGX^®^, to commonly consumed foods on glycemic response, glycemic index and GRIP: a simple and effective strategy for reducing post prandial blood glucose levels - a randomized, controlled trial

**DOI:** 10.1186/1475-2891-9-58

**Published:** 2010-11-22

**Authors:** Alexandra L Jenkins, Veronica Kacinik, Michael Lyon, Thomas MS Wolever

**Affiliations:** 1 Glycemic Index Laboratories, Inc, Toronto, ON, M5C 2N8; 2 Canadian Centre for Functional Medicine, Coquitlam, BC, V3K 6Y2; 3 Food, Nutrition and Health Program, University of British Columbia, BC, V6T 1Z4, Canada

## Abstract

**Background:**

Reductions in postprandial glycemia have been demonstrated previously with the addition of the novel viscous polysaccharide (NVP), PolyGlycopleX^® ^(PGX^®^), to an OGTT or white bread. This study explores whether these reductions are sustained when NVP is added to a range of commonly consumed foods or incorporated into a breakfast cereal.

**Methods:**

Ten healthy subjects (4M, 6F; age 37.3 ± 3.6 y; BMI 23.8 ± 1.3 kg/m^2^), participated in an acute, randomized controlled trial. The glycemic response to cornflakes, rice, yogurt, and a frozen dinner with and without 5 g of NVP sprinkled onto the food was determined. In addition, 3 granolas with different levels of NVP and 3 control white breads and one white bread and milk were also consumed. All meals contained 50 g of available carbohydrate. Capillary blood samples were taken fasting and at 15, 30, 45, 60, 90 and 120 min after the start of the meal. The glycemic index (GI) and the glycemic reduction index potential (GRIP) were calculated. The blood glucose concentrations at each time and the iAUC values were subjected to repeated-measures analysis of variance (ANOVA) examining for the effect of test meal. After demonstration of significant heterogeneity, differences between individual means was assessed using GLM ANOVA with Tukey test to adjust for multiple comparisons.

**Results:**

Addition of NVP reduced blood glucose response irrespective of food or dose (p < 0.01). The GI of cornflakes, cornflakes+NVP, rice, rice+NVP, yogurt, yogurt+NVP, turkey dinner, and turkey dinner+NVP were 83 ± 8, 58 ± 7, 82 ± 8, 45 ± 4, 44 ± 4, 38 ± 3, 55 ± 5 and 41 ± 4, respectively. The GI of the control granola, and granolas with 2.5 and 5 g of NVP were 64 ± 6, 33 ± 5, and 22 ± 3 respectively. GRIP was 6.8 ± 0.9 units per/g of NVP.

**Conclusion:**

Sprinkling or incorporation of NVP into a variety of different foods is highly effective in reducing postprandial glycemia and lowering the GI of a food.

**Clinical Trial registration:**

NCT00935350.

## Background

Epidemiological evidence suggests that postprandial glucose levels have a stronger relationship with cardiovascular events than fasting glucose in individuals with diabetes [1,2]. Recent attention has therefore focused on dietary strategies which target the reduction of postprandial hyperglycemia both as a treatment and possible prevention of diabetes and cardiovascular disease. Both quantity and quality of ingested carbohydrate can affect post prandial glucose levels [3,4]. A meta analysis by Livesey et al [5] which investigated the relation between dietary glycemic properties and health outcomes, concluded that reductions in the GI of the diet do improve glycemic control but that unavailable carbohydrate intake is equally important. Soluble viscous fibers are unique in that incorporation into the diet would both increase the unavailable carbohydrate intake and, in effect, lower the GI of the diet by their ability to flatten the postprandial glycemia [6,7].

Increasing viscous fiber consumption however is hampered by both palatability issues and variability in effectiveness[8]. The higher the viscosity of the fiber, the greater its effect on postprandial glycemia [6,9,10], however it is the viscosity of the fiber that decreases palatability and prohibits practical application of soluble fiber in the diet. Processing and mode of administration are known to alter its effectiveness; isolation of beta glucan using two different processes, demonstrated greater reductions with the beta-glucan product in which the viscosity had been retained [11]. Consumption of psyllium between meals over two weeks had no effect of blood cholesterol levels in modestly hypercholsterolemic individuals, however consuming the same dose of psyllium when mixed with the food resulted in significant decreases in total and LDL cholesterol levels [12].

Recently, a commercial novel viscous polysaccharide complex has been developed which is marketed in both the USA and Canada under the trade name PolyGlycopleX^® ^(PGX^®^) (InovoBiologic Inc, Calgary, AB, Canada). This complex consists of three viscous non starch polysaccharides which are processed using proprietary technology (EnviroSimplex^®^) which delays the onset of viscosity and thus increases palatability of the fiber when added to food. Previously, we reported the glucose lowering effect of this fiber in a dose responsive manner when mixed with a glucose drink and sprinkled onto a standard white bread demonstrating that the processing did not interfere with the effectiveness of the fiber [13]. From this study the reduction in glycemic index units per gram of fiber (GRIP) was calculated to be 7 GI units per gram of NVP when added to a solid food. Where GRIP is defined as the change in the GI value (or units) of the food caused by the addition of fiber and divided by the number of grams of fiber used to effect this change. However it is not known whether this relationship would be maintained when the NVP was added to a range of commonly eaten foods. This study therefore evaluates the effect of the addition or incorporation of NVP into a range of foods on postprandial glycemia and whether the reductions in glycemic index could be predicted using the GRIP factor established previously.

## Methods

### Study participants

Ten healthy subjects (4M, 6F; age, 37.3 ± 3.6 y; BMI 23.8 ± 1.3 kg/m^2 ^participated in an acute randomized, controlled study. Subjects were recruited from the Glycemic Index Laboratories clinic volunteer roster. Entry criteria included BMI < 35 kg/m^2 ^and fasting blood glucose < 5.5 mmol/L, subjects taking medications or dietary supplements were excluded. The study was approved by the Western Institutional Review Board (Washington, DC) and conducted in compliance with HIPAA guidelines. Informed written consent was obtained from all subjects prior to the start of the study. Subjects received a financial reward for their participation.

### Study Design

The study design was an open label, randomized study with fifteen treatments taken by each subject group. Each subject underwent treatments on separate days, with each subject performing up to 2 tests per week with at least one day between tests. On each test day, subjects came to Glycemic Index Laboratories, Inc. in the morning after a 10-14 h overnight fast. After being weighed and having a fasting blood sample obtained by finger-prick, the subject then consumed a test meal within 10 minutes, and further blood samples were obtained at 15, 30, 45, 60, 90 and 120 minutes after the start of the test meal.

### Fiber Supplement

The NVP used in this study is marketed under the trade name PGX^® ^(InovoBiologic Inc, Calgary, Alberta, Canada) and is manufactured from konjac-xanthan gum and sodium alginate using a proprietary process (EnviroSimplex^®^) to create a novel polysaccharide complex which exceeds the viscosity of the constituent ingredients. PGX^® ^is 87.4% dietary fiber, of which 81.8% is soluble. A typical recommended serving ranges from 2.5 to 5 grams depending on the application.

### Study Meals

A total of 11 test meals were consumed (table 1). All test meals contained at least 50 g of available carbohydrate and consisted of Corn Flakes^® ^(Kellogg Canada) with 125 mL milk, Jasmine long grain rice, turkey dinner (Roast Turkey Dinner, Stouffer's), strawberry yogurt (Astro Yogurt) with or without 5 g of NVP sprinkled onto it. In addition three different granolas were also consumed into which either 0 g, 2.5 g or 5 g of NVP had been incorporated. To control for the additional carbohydrate from the milk taken with the cornflakes, an additional control white bread was consumed with 125 ml of 2% butterfat milk. Finally, all subjects also consumed the control white bread on three separate occasions to allow calculation of the glycemic index of the meals (table 1). The white bread was baked in a bread maker in loaves containing 250 g available carbohydrate. The ingredients for each loaf (250 ml warm water, 340 g all purpose flour (Robin Hood, Markham, Canada), 7 g sugar, 4 g salt and 6.5 g dry yeast) were placed into a bread maker according to instructions, and the machine turned on. After the loaf had been made, it was allowed to cool for an hour, and then weighed and after discarding the crust ends, the remainder was divided into portion sizes containing 50 g available carbohydrate. These portions were frozen prior to use, and reheated in the microwave prior to consumption. Subjects were also given a choice of 1 or 2 cups of either water, tea or coffee with or without 25 ml 2% butterfat milk. The beverage consumed by each subject remained the same on each test day. All test meals were given in random order.

**Table 1:** Amount, energy and macronutrient profile of meals: The control white bread meal was repeated on 3 separate occasions.

Test Meal	Amount (g)	Energy (kcal)	Protein (g)	Fat (g)	Total CHO (g)	Dietary Fibre (g)	Available CHO (g)	GI*
**Control White Bread**	104	244	7.6	1.6	52.8	2.8	50	71

**White Bread** **+ 2% milk**	104 +125 ml	313	7.6 +6.0	1.6 +2.4	52.8 +6	2.8 +0	50 +6	71

**Cornflakes** **+ 2% milk**	60 +125 ml	286	4 +6.0	0 +2.4	52 +6	2 +0	50 +6	79

**Rice**	62.5	217	4.2	0	50.0	0	50	70

**Turkey Dinner**	422	388	21.6	11.3	55.4	5.6	50	NA

**Fruit Yogurt**	250	268	8	4	50	0	50	47

**Blueberry Granola** **(control)**	92	369	9.3	14.6	62.3	12.3	50	67

**Blueberry Granola** **(2.5 g PGX)**	93	369	10.0	14.3	63.4	13.4	50	NA

**Blueberry Granola** **(5 g PGX)**	98	378	10.4	15.2	65.8	15.8	50	NA

The Cornflakes, rice, yogurt and turkey dinner meals were consumed two times: once with and once without 5 g of NVP. There were three different granola cereals containing either 0, 2.5 or 5 g of NVP. The white bread and milk meal was consumed to balance the extra carbohydrate in the cornflake and milk meal. Values are derived from direct analysis (white bread), standard food tables or the manufacturer. *GI values are mean values reported in the literature (20).

### Statistical Analysis

The results are expressed as means ± SEM. The mean blood glucose results of the 3 white breads were used in calculating the glycemic index. The incremental areas under the blood glucose response curves, ignoring the area below the fasting level, were calculated geometrically [14]. The blood glucose area under the curve for each food was expressed as a percent of the mean area for the corresponding control tests and the resulting mean values for all subjects represented the GI of the food. The mean of the resulting values was the food GI based on the glucose scale where the glycemic index of bread is 71 and glucose 100. The blood glucose concentrations at each time and the iAUC values were subjected to repeated-measures analysis of variance (ANOVA) examining for the effect of test meal. After demonstration of significant heterogeneity, the significance of the differences between individual means was assessed using GLM ANOVA with Tukey test to adjust for multiple comparisons. In addition, the postprandial glucose response to the paired meals was also compared using the same statistical analysis. To calculate the Glycemic Reduction Index Potential (GRIP) of NVP, the GI change for each individual dose was averaged and calculated per gram of NVP.

## Results

### Sprinkling

**Figure 1:**
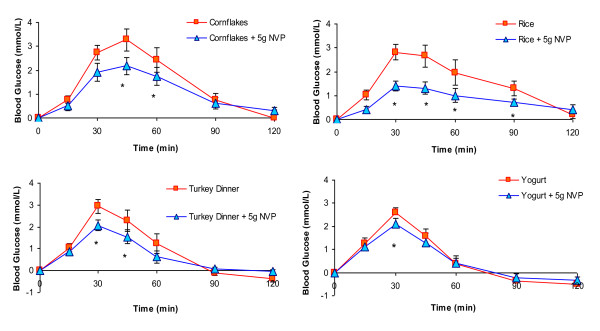
**Effect of adding 5 g of a novel fiber to commonly consumed meals on postprandial glycemia**: Incremental postprandial blood glucose responses after cornflakes, rice, roast turkey dinner or fruit yogurt with or without 5 g of NVP. Data are expressed as Mean ± SEM, *significant difference in incremental blood glucose levels (p < 0.05).

Sprinkling of 5 g NVP on the cornflakes, rice, yogurt and turkey dinner meals significantly reduced the blood glucose response irrespective of type of food or dose (p < 0.01). Comparison of the meals with and without NVP, showed that addition of NVP to the meals significantly reduced incremental postprandial glucose levels at 30, 45, and 60 min after consumption of the rice meal and at 45 min after both the cornflakes and turkey dinner meals (p < 0.05, Figure 1). NVP significantly reduced the incremental areas after the cornflakes (184.9 ± 28.5 vs. 132.7 ± 23.5 mmol.min/L, p < 0.02), and rice (185.6 ± 31.7 vs. 98.8 ± 17.1 mmol.min/L, p < 0.001) but not after the turkey dinner (126.8 ± 22.5 vs. 89.1 ± mmol.min/L) or yogurt and yogurt+NVP meals (92.4 ± 11.8 vs 84.9 ± 14.5 mmol.min/L). There were no significant differences in incremental blood glucose areas between the mean of the white bread controls (WB) and the white bread and milk (WB+M) test meal (158.9 ± 20.7 vs 159.3 ± 28.2 mmol/L.min).

### Granola

**Figure 2:**
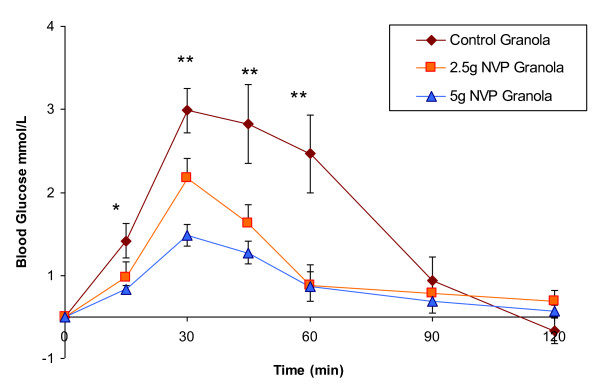
**Effect of incorporating a novel fiber into granola on postprandial glycemia**: Incremental postprandial blood glucose responses after a control blueberry granola (0 g of NVP), or a blueberry granola with 2.5 or 5 g of NVP incorporated. All meals contained 50 g of available carbohydrate. Data are expressed as Mean ± SEM; *significantly different from control granola (p < 0.05).

At 15, 30, 45 and 60 min blood glucose levels of the granolas containing NVP were significantly lower when compared to control granola (Figure 2). Incremental areas of the 2.5 g granola (86.6 ± 15.3 mmol.min/L) and the 5 g NVP blueberry granola (50.3 ± 10.1 mmol.min/L) were significantly reduced when compared to the control granola (147.7 ± 25.7 mmol.min/L, p < 0.0001).

### Glycemic Index and GRIP

The GI of cornflakes, cornflakes+NVP, rice, rice+NVP, yogurt, yogurt+NVP, turkey dinner, and turkey dinner+NVP were 83 ± 8, 58 ± 7, 82 ± 8, 45 ± 4, 44 ± 4, 38 ± 3, 55 ± 5, 41 ± 4, respectively. The GI of the control granola (0 g NVP), and the granolas with 2.5 g and 5 g of NVP were 64 ± 6, 33 ± 5, and 22 ± 3 respectively. Percent reductions in GI of the cornflakes, rice, turkey dinner and yogurt meals with NVP were 26%, 45%, 24% and 12% respectively when compared to the meals without NVP. Percent reductions for the 2.5 g NVP blueberry granola and 5 g NVP blueberry granola were 46% and 64% respectively when compared to the control granola. The average GRIP of all meals was 6.8 ± 0.9 GI units per g of NVP.

## Discussion

This study demonstrates that this particular viscous fiber preparation, PGX^®^, is effective in lowering postprandial glycemia when either sprinkled or incorporated into commonly eaten foods and confirms earlier predictions that the GI of a food would be reduced.

Enrichment of the diet using soluble fiber is particularly attractive as it both increases the dietary fiber levels of the diet as is recommended by health agencies such as the American and Canadian Diabetes Associations and the American Dietetic Association [15-17] and it has the further benefit of lowering the GI of the diet with its accompanying potential for health benefits [18]. A recent meta-analysis published in the American Journal of Clinical Nutrition by Barclay et al [19] found significant positive relationships between highest and lowest quartiles of GI and glycemic load and incidence of type 2 diabetes, coronary heart disease, gallbladder disease, and breast cancer. The protection associated with low GI or low GL diets were comparable to that seen with whole grain or high fiber intake [19]. The results of this analysis again support the premise that reducing postprandial blood glucose excursions may be an important strategy to reduce development of certain chronic diseases. The NVP reduced the glycemic index of the cornflakes, rice, turkey dinner, and strawberry yogurt by 26%, 45%, 24% and 12% respectively. Using the classification for Brand-Miller et al (20), where low GI foods are defined as having a GI less than 55, medium greater than 55 and less than 70 and high GI foods as greater than 70, many of the foods could be reclassified with respect to their GI. Cornflakes was changed from a high GI food to a medium GI food, the rice from a high GI food to low GI food, the turkey dinner and the granolas from a medium GI food to a low GI food. The yogurt was already a low GI food, but addition of NVP induced a further, although modest, 12% reduction in GI. The ability of viscous fiber to lower postprandial glycemia has long been known [6,21] however its effect has always been dependent on being intimately mixed with the carbohydrate in the food [22,23]. Sprinkling of the fiber usually results in clumping and a loss of effect [24] and therefore necessitates more intensive food preparation if the effect is to be preserved. The results of this study confirms that incorporation of the fiber into the meal is still more effective than sprinkling as was illustrated by larger percent reductions seen with the granolas. Nevertheless, unlike unprocessed viscous fibers, significant reductions in postprandial glycemia were observed when the NVP was sprinkled onto the food. This property will allow greater flexibility of its use for the consumer.

To allow a direct comparison of the effectiveness of different substances in affecting the GI of a food, we proposed the term "GRIP" (Glycemic Reduction Index Potential) concept (CMJ). Previous studies estimated the GRIP of NVP to be 5 when added to liquids and 7 when added to a solid food [13,25]. This study again confirmed that, on average the NVP reduced the GRIP by 7 units per gram of fiber and compares favorably with a fiber such as oat bran which has an estimated GRIP of 4 units per gram of fiber [26]. As a consequence of the high GRIP factor of NVP, smaller quantities can be used to give equivalent results to other viscous fibers, doses of which ranged from 8-15 g [6,27,28], compared to the 5 g used in this study.

Limitations of this study include the relatively small number of meals and subjects tested and the acute nature of this study. Further studies need to be undertaken to confirm consistent effects across a wider range of foods, and whether the acute reductions seen translate in flattening of the fluctuations seen in glycemia during the day.

## Conclusions

In conclusion, use of a soluble dietary fiber supplement may be an effective means of increasing the dietary fiber intake as recommended for individuals and simultaneously reduce the GI of the diet. However, reduced palatability of the high fiber foods and the large quantities needed to confer a health benefit are often barriers to increasing consumption. This particular NVP preparation has an advantage over most fibers in that it is a standardized product with a consistently high viscosity which allows a reduction in the amounts required. It has a powerful GRIP value with respect to glucose lowering effect of high GI foods, which is either equivalent or higher than other fiber preparations. The use of this fiber may potentially have positive health implications and further research is therefore warranted to confirm the long term health benefits of this novel viscous polysaccharide.

## Competing interests

ALJ received financial remuneration for the preparation of the manuscript and is a consultant to the parent company of the sponsor.

VK was an employee of InovoBiologic Inc. during the planning and execution of these studies.

MRL is a consultant to the parent company of the sponsor.

TMSW - no competing interests

## Authors' contributions

ALJ, VK, and MRL participated in the design of the study. ALJ and TW performed the statistical analysis. ALJ coordinated the research and drafted the manuscript. All authors read and approved the final manuscript.
